# The First Complete Mitochondrial Genome of Lachninae Species and Comparative Genomics Provide New Insights into the Evolution of Gene Rearrangement and the Repeat Region

**DOI:** 10.3390/insects12010055

**Published:** 2021-01-11

**Authors:** Hui Zhang, Qian Liu, Congcong Lu, Jun Deng, Xiaolei Huang

**Affiliations:** State Key Laboratory of Ecological Pest Control for Fujian and Taiwan Crops, College of Plant Protection, Fujian Agriculture and Forestry University, Fuzhou 350002, China; zhanghui1903@163.com (H.Z.); liuqian9502@163.com (Q.L.); lcchuaer@163.com (C.L.); tiancai1282008@126.com (J.D.)

**Keywords:** mitogenome, aphid, repeat region, gene rearrangement

## Abstract

**Simple Summary:**

The Lachninae is a unique aphid lineage with some particular characteristics. The first complete mitochondrial genome from this subfamily is reported here, with *Stomaphis sinisalicis* as a representative. The gene arrangement of the new mitogenome differs greatly from the normal gene order of other aphids and insects. The existence of a tandem repeat region in the Lachninae mitogenome may suggest it is probably an ancient feature of aphid mitogenomes. Phylogenetic analysis indicates a basal position of Lachninae within Aphididae.

**Abstract:**

Complete mitochondrial genomes are valuable resources for different research fields such as genomics, molecular evolution and phylogenetics. The subfamily Lachninae represents one of the most ancient evolutionary lineages of aphids. To date, however, no complete Lachninae mitogenome is available in public databases. Here we report the *Stomaphis sinisalicis* mitogenome, representing the first complete mitogenome of Lachninae. The *S. sinisalicis* mitogenome is consist of 13 protein-coding genes (PCGs), two rRNA genes (rRNAs), 22 tRNA genes (tRNAs), a control region and a large tandem repeat region. Strikingly, the mitogenome exhibits a novel, highly rearranged gene order between *trnE* and *nad1* compared with that of other aphids. The presence of repeat region in the basal Lachninae may further indicate it is probably an ancestral feature of aphid mitogenomes. Collectively, this study provides new insights on mitogenome evolution and valuable data for future comparative studies across different insect lineages.

## 1. Introduction

Complete mitochondrial genomes of insects are highly conserved circular molecules, which are valuable resources for investigating the molecular evolution, phylogenetic relationships and gene arrangement among different lineages, due to their rare recombination, rapid evolutionary rate and evolutionary conservation in organization [1,2,3]. The double-stranded molecules are generally composed of 13 protein coding genes (PCGs), two ribosomal genes (rRNAs), 22 tRNA genes (tRNAs) and a large non-coding control region (also called AT-rich region). In some taxa, such as some hemipteran aphid species, however, there is an additional unique non-coding tandem repeat region comprising different repeat units and copy numbers [4,5,6,7]. The gene order within insect mitogenomes is basically pretty conservative and identical to that of proposed Insecta ancestral gene arrangement pattern, with some exceptions of gene rearrangements involving tRNAs, rRNAs or PCGs [1,8,9,10,11,12]. Hemipteroids are one of the most distinct examples having a highly rearrangement frequency among insects, including species from orders Phthiraptera, Psocoptera, and Thysanoptera [1,13]. High rearrangement rates are also found in some Hemipterans including planthoppers (Delphacida), bugs (Aradidae, Enicocephalidae, Largidae, Pyrrhocoridae), whiteflies (Aleyrodidae), and more recently, scale insects (Coccoidea) [1,14,15,16,17,18]. While very few gene rearrangements are found in other hemipteran taxa such as aphids, those gene arrangement patterns remain to be confirmed by investigating more taxa. Mitogenome arrangement data have been supposed to provide useful information for investigating evolutionary relationships and insect evolutionary history because gene rearrangement of typical 37 genes in mitogenome is extremely uncommon and unlikely to replicate through convergent evolution [2,3]. With the growing availability of complete mitogenomes of insects, comprehensive comparative studies across different lineages should promote our understanding for their molecular evolution and phylogenetic relationships. However, mitogenomes of some insect taxa are still underrepresented.

Aphids are an important and diverse group within Hemiptera that includes many plant pests with complex life cycles and a large number of host plants. In the Aphididae, more than 5000 aphid species from 24 subfamilies have been described [19]. Of the thousands of extant aphid species, complete mitochondrial genomes are known for only 43 species from six subfamilies (Table S1). For some major aphid lineages, not even a single complete mitochondrial genome has been reported. The limited mitogenomic resources and small sampling has hampered widely comparative studies for the mitogenome sequences, gene arrangements and molecular evolution among aphids. Moreover, an extensive comparative analysis among aphids will favor gaining insights into the evolution of some unique characteristics for aphid mitogenomes, such as the presence of a large tandem repeat region. The unique tandem repeat region in the aphid mitogenomes is generally locating between the *trnE* and *trnF* but differ largely in length and copy number of repeat units [5,7]. This repeat region was first reported in several species from the subfamily Aphidinae and was once considered to be a lineage-specific feature which occurred from independent evolutionary events [20,21], whereas several species from other subfamilies have subsequently been found to contain a repeat region as well. So far, 18 aphid species from four subfamilies (Aphidinae, Eriosomatinae, Greenideinae and Hormaphidinae) have been reported to contain this tandem repeat region [4,22,23,24]. It was proposed that the repeat region in aphid mitogenomes either originated from the recent common ancestor of aphids followed by subsequent loss events throughout aphid diversification or independently evolved among different aphid lineages [5,6,25]. The former hypothesis has been increasingly accepted recently as more species from other subfamilies have been found to contain this region, and high sequence similarity of this region among species has been discovered [25]. However, there has been no data from a more ancient aphid lineage with repeat region to support this hypothesis, which will help to better understand the origin and evolution of repeat region in aphid mitogenomes.

**Figure 1:**
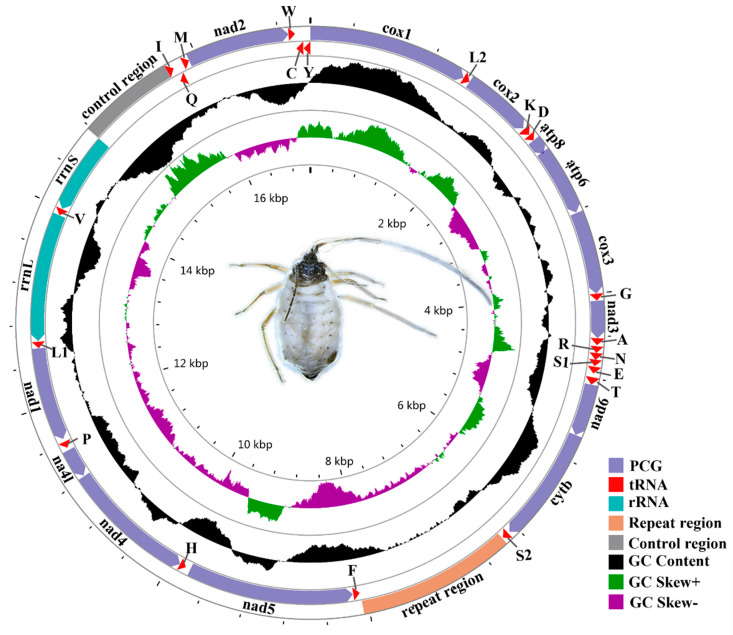
Circular map of the complete mitogenome of *Stomaphis sinisalicis*. Different colors indicate different types of genes and regions. Genes shown at the outer circle are located on the J strand, and those at the inner circle are located at the N-strand.

The Lachninae represent an important and unusual aphid lineage with complicated host associations, encompassing distinct species feeding on both angiosperm and gymnosperm hosts [26]. This subfamily has been supposed to be one of the most ancient clades in the Aphididae according to several recent phylogenetic studies [27,28]. Up to now, however, no complete mitochondrial genome from Lachninae has been deposited in the GenBank (http://www.ncbi.nlm.nih.gov; accessed on October 19, 2020). *Stomaphis sinisalicis* is one representative species of the aphid genus *Stomaphis*, which represent a unique evolutionary lineage in the aphid subfamily Lachninae and are known for their large body size and particularly long rostrum (see the aphid in Figure 1) [29,30]. *S. sinisalicis* is mainly distributed in northern China with temperate climate, including Beijing, Hebei, Liaoning, Ningxia and Shandong. This species inhabits and feeds on the base of tree trunk or crevices of thick bark of *Salix* spp. and *Populus* sp., and generally establishes close mutualistic relationship with ants [29].

In this study, we generated and analyzed the first complete mitochondrial genome for *S. sinisalicis*. The structural organization, nucleotide composition, codon usage and gene order arrangement were analyzed and compared against that of well characterized complete mitogenomes of other aphids to investigate the unique features and mitogenome evolution of aphids. This complete mitogenome reported here contributes to aphid higher phylogeny reconstruction based on mitogenome sequences and promotes future comparative mitogenome studies, which should help understand the mitogenome evolution and gene arrangement pattern across different aphid lineages.

## 2. Materials and Methods

### 2.1. Sample Preparation and DNA Extraction

The *S. sinisalicis* samples used in this study were collected on *Salix babylonica* from Beijing Olympic Forest Park, China (40°1′30.8″ N, 116°23′48.4″ E), in June 2018. All collected samples and voucher specimen (20180615-12) were preserved in 95% ethanol under −80 °C and deposited in the Insect Systematics and Diversity Lab at Fujian Agriculture and Forestry University, Fuzhou, China. Samples of *S. sinisalicis* were morphologically identified based on previous morphological descriptions of this species by the corresponding author.

### 2.2. Mitogenome Sequencing and Assembly

The next-generation sequencing was performed at the Biomarker Technologies Co., Ltd. (Beijing, China). For library construction, genomic DNA concentration was detected using ExKubit dsDNA HS test kit (ExCell Biotech Co., Ltd., Shanghai, China) with a Qubit fluorimeter (Invitrogen, Carlsbad, CA, USA). The qualified high-quality genomic DNA was used for next-generation sequencing subsequently. For library construction, TrueLib DNA Library Rapid Prep Kit for Illumina (ExCell Biotech Co., Ltd.) was used with 250 ng DNA as template, then sequencing was conducted using the NovaSeq 6000 System (Illumina, San Diego, CA, USA) (2 × 150 bp paired-end reads) with an insert size of 350 bp. Finally, approximately 10.4 Gb raw data was obtained and further filtered to remove adapters and low-quality reads, including reads with an N base content reached 10% of the read length and those with low quality bases (quality value ≤ 10) over 50% of the read length. Mitogenome was assembled using a total of 7.1 Gb clean data (clean reads: 23,773,262) by NovoPlasty v. 2.7.1 [31] with default parameters, using a COI sequence from *S. sinisalicis* as the seed sequence. The final genome assembly showed a high coverage and depth (Figure S1).

### 2.3. Annotation and Analysis of S. sinisalicis Mitogenome

The assembled mitogenome was first annotated using MITOS2 web server (http://mitos2.bioinf.uni-leipzig.de/index.py) [32] with the invertebrate mitochondrial genetic code. The boundaries of 13 PCGs and two rRNA genes were further determined by alignment with the known corresponding sequences of closely related aphid species. All tRNA genes in *S. sinisalicis* were identified using the MITOS2 web server and program ARWEN v. 1.2 [33], and their clover-leaf secondary structure were further drawn by RNAplot integrated in the ViennaRNA Package [34]. Tandem repeat region was detected by using the Tandem Repeats Finder Web server (http://tandem.bu.edu/trf/trf.html) [35]. The AT-rich control region was identified via boundaries of adjacent genes. Circular maps of the assembled mitogenome of *S. sinisalicis* was finally drawn with CGView Server [36]. MEGA7 software [37] was used to analyze the nucleotide composition and relative synonymous codon usage (RSCU). To better understand the base usage bias of the mitogenome, we also calculated the nucleotide skew values using the formulae AT-skew = (A − T)/(A + T) and GC-skew = (G − C)/(G + C) [38].

### 2.4. Phylogenetic Analysis

For phylogenetic analysis, all 13 PCG sequences from *S. sinisalicis* were collated to that of 24 available mitogenomes of representative species from different subfamilies of Aphididae and that of *Adelges tsugae* (Adelgidae) as ourgroup (Table 1). All PCG sequences (excluding the stop codons) of these 26 aphid species was aligned individually using codon-based multiple alignments by MAFFT v7.149 software [39] with default settings. After removing ambiguously aligned sites, alignments of 13 aligned PCGs were concatenated into a single alignment dataset using PhyloSuite v1.1.15 [40]. The best-fit substitution model was determined using the ModelFinder [41] integrated in IQ-TREE [42], and GTR+F+R4 was inferred as the optimal model according to the Bayesian Information Criterion (BIC) and was then used for subsequent phylogenetic analysis. Maximum-likelihood (ML) phylogenetic tree was reconstructed with the *A. tsugae* as the outgroup using IQ-TREE with 1000 ultrafast bootstrapping replicates. We also repeated the phylogenetic reconstruction with the dataset partitioned by each PCG, which yielded phylogenetic tree with identical topological relationships (Figure S2).

### 2.5. Comparative Mitogenomic Analysis

The base composition, gene length, and genomic organization of all available aphid complete mitogenomes were collected and calculated for comparative genomic analysis. Furthermore, the online Interactive Tree of Life (iTOL) v 4 tool [61] was used for gene order visualization of known aphid mitogenomes, with the gene arrangement pattern mapping onto an inferred phylogenetic tree. We also detected the putative gene rearrangement events occurred in mitogenomes of each aphid species using the web-based program Common interval Rearrangement Explorer (CREx) [62] with arrangement of *Drosophila yakuba* as the inferred ancestral gene order [12,63]. And the genome rearrangement scenarios including transpositions, reverse transpositions, reversals, and tandem duplication random loss (TDRL) were reconstructed between *D. yakuba* and each aphid.

## 3. Results and Discussion

### 3.1. Mitogenome Features of S. sinisalicis and Other Aphids

**Figure 2:**
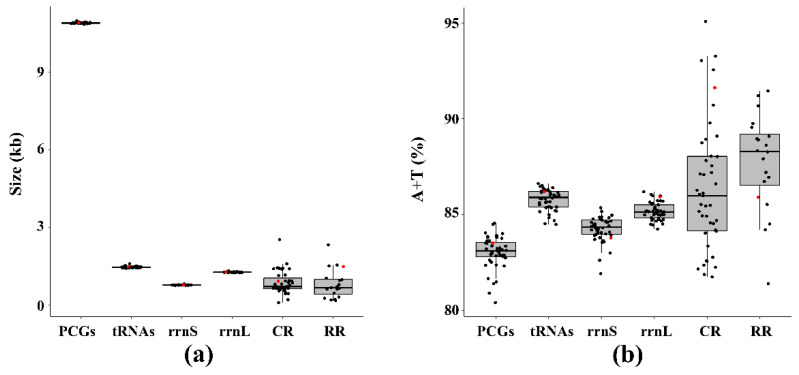
The size (**a**) and AT content (**b**) of PCGs, *rrnL*, *rrnS*, CR and RR of 44 aphid species with the red dots representing the *Stomaphis sinisalicis*. Lower edge of the gray rectangle, 25 percentile; central black bar within the rectangle, median; upper edge of the rectangle, 75 percentile.

Known complete mitogenomes of aphids range from 15,049 (*Appendiseta robiniae*) to 17,954 bp (*Aphis glycines*) (Table 1). The mitochondrial genome of *S. sinisalicis* was 17,109 bp in length (the circular map is shown in Figure 1), longer than most other known aphid mitogenomes (Table 1). As in the case in other insects, the *S. sinisalicis* mitogenome contained the typical 13 PCGs, two rRNAs and 22 tRNAs, in which 14 tRNAs and 9 PCGs were transcribed on the majority strand (J-strand), while the others (eight tRNAs, two rRNAs and four PCGs) were located on the minority strand (N-strand) (Table 2). Besides, the *S. sinisalicis* mitogenome also contained a typical AT-rich control region locating between *rrnS* and *trnI*, and a large non-coding tandem repeat region. For aphid mitogenomes, the sizes of control region and repeat region vary greatly among different species, whereas the PCGs, tRNAs and two rRNAs show little variation in length (Figure 2a), suggesting that the mitogenome size of different aphids is largely determined by the size of non-coding regions.

Eleven gene overlaps with a total of 43 bp were present in *S. sinisalicis* mitogenome, ranging from 1 to 20 bp, with the longest overlap (20 bp) occurring between *atp6* and *atp8* (Table 2). The overlap between *atp8*-*atp6* is also found in mitogenomes of other arthropods [7,64]. Moreover, there were also 19 intergenic spacers between genes, with the longest intergenic spacer (50 bp) existing between *nad5* and *trnH*. Longer intergenic regions were also found in the junctions between *trnE*–*trnT* (34 bp) and *trnQ*–*trnM* (35 bp) (Table 2).

The *S. sinisalicis* mitogenome contained an obviously higher A+T content (84.8%) and a lower G+C content (15.2%). These values are comparable to that of other aphid species, which have an average A+T content of 84.0%, varying from 81.7% (*A. robiniae*) to 85.7% (*Greenidea ficicola*) (Table S1). This A+T bias in *S. sinisalicis* mitogenome was reflected in all components of its genome. The A+T content of control region and repeat region were 91.5% and 85.9%, and the PCGs, tRNAs and rRNAs had an average A+T content of 83.5%, 86.4% and 85.1%, respectively (Table 3). In aphids, the A+T content varies greatly for the repeat region and control region, but a small variation for PCGs, tRNAs and two rRNAs (Figure 2b), indicating that higher A+T contents in the control region and repeat region probably raise the total A+T content of aphid mitogenomes.

**Figure 3:**
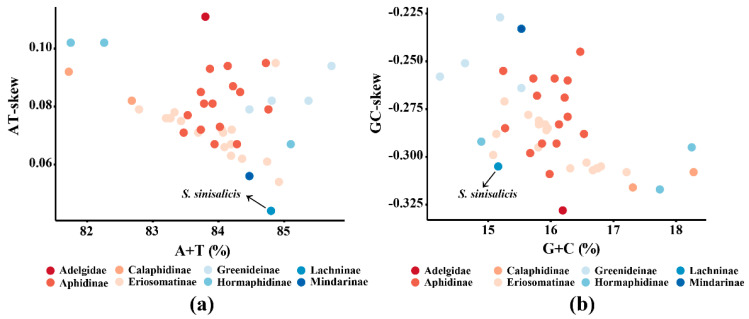
Nucleotide composition of *Stomaphis sinisalicis* and 44 others complete aphid mitogenomes (including the *Adelges tsugae*). (**a**) The A+T content and AT skew. (**b**) The G+C content and GC-skew. See Table S1 for details of aphid species.

The nucleotide skew analysis showed that the whole *S. sinisalicis* mitogenome exhibit a positive AT-skew (0.044) and a negative GC-skew (−0.305). A similar pattern has been found in other aphid mitogenomes [4], with the AT-skew ranging from 0.054 in *Paracolopha morrisoni* to 0.102 in *Hamamelistes spinosus* and a GC-skew varying from −0.317 in *Hormaphis betulae* to −0.227 in *Cervaphis quercus*. These results indicate that *S. sinisalicis* mitogenome has a weakest A skew and a stronger C skew in compared to other known aphid mitogenomes (Figure 3a,b). This base composition bias has been suggested to be associated with replication and transcription of mitochondrial genome [65].

### 3.2. Gene Arrangement Patterns

**Figure 4:**
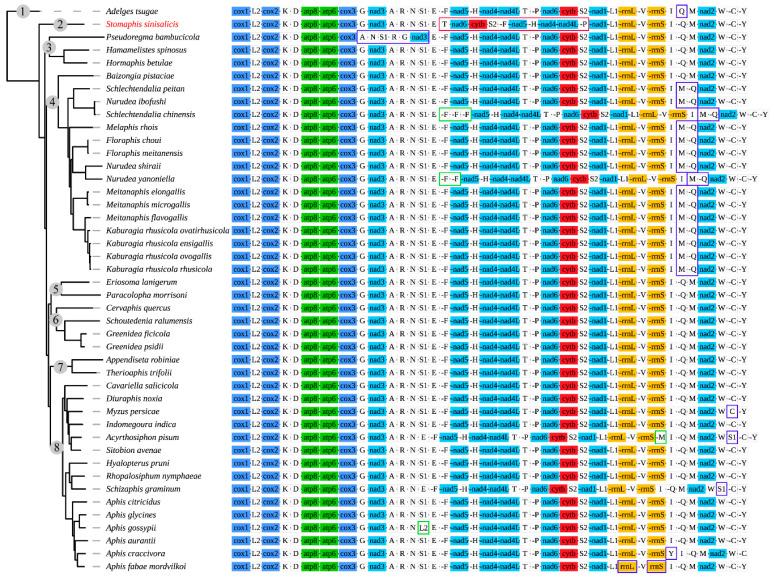
Gene arrangement in mitochondrial genomes of aphids (including *Adelges tsugae*). Numbers labeled on clades of the left phylogenetic tree indicate different family, subfamily or tribe of aphids, which correspond to clades in the phylogenetic tree below, 1: Adelgidae; 2: Lachninae; 3: Hormaphidinae; 4: Fordini (Eriosomatinae); 5: Eriosomatini (Eriosomatinae); 6: Greenideinae; 7: Calaphidinae; 8: Aphidinae. Within the right gene arrangement image, the color blocks represent different categories of genes, dark blue: cytochrome oxidase complex; turquoise: NADH dehydrogenase complex; red: cytochrome b; ginger: ribosomal RNA genes; grey white: transfer RNA genes. The transfer RNA genes are indicated by the corresponding capital letters. Putative gene rearrangement events identified by CREx analysis (with the *Drosophila yakuba* as referenced ancestral gene arrangement) are marked by different color boxes. The predicted tandem-duplication-random-loss events (TDRL) occurred in *Stomaphis sinisalicis* are indicated with red box; the other gene rearrangement events (including the transposition, inversion, inverse transposition) are indicated by blue box; gene duplications are labeled with green boxes. Non-coding region was excluded from the analysis.

Gene arrangement of mitogenomes has been supposed to be quite conservative among insects and identical to that of the ancestral pancrustacean mitogenomes [1,12,66]. Various gene rearrangements have been observed in some insect taxa, including major rearrangement involving the PCGs and rRNAs, as well as minor rearrangement involving only tRNAs [1]. Mitochondrial genome rearrangement is rarely reported among aphids, and most of them have a gene order resembling the ancestral insect arrangement. However, in *S. sinisalicis* mitogenome, the gene order between *trnE* and *nad1* was highly rearranged, with the order of gene cluster *trnF*-*nad5*-*trnH*-*nad4*-*nad4l*-*trnT*-*trnP*-*nad6*-*cob*-*trnS2* shifting into the *trnT*-*nad6*-*cob*-*trnS2*-*trnF*-*nad5*-*trnH*-*nad4*-*nad4l*-*trnP* (Figure 4). This gene arrangement pattern differed greatly from that of other aphid species, which has been thought highly conserved within Hemipteran insects and similar to the ancestral gene order [4,15,63].

Rearrangement of mitogenomes can generally be classified into several gene movements: transposition, inversion, and inverse transposition [66]. Several models have been proposed to explain the rearrangement of mitogenome genes, such as the tandem duplication random loss (TDRL) model [67,68], tandem duplication/non-random loss (TDNL) model [69], recombination model [70] and tRNA mispriming model [71]. According to the results of CREx analysis, the rearrangement event occurred in *S. sinisalicis* mitogenome can be better explained by the widely accepted tandem duplication random loss (TDRL) model [67,68]. Based on this model, the gene cluster locating *trnE* and *nad1* in *S. sinisalicis* mitogenome can be separated into four gene blocks: *trnF*-*nad5*-*trnH*-*nad4*-*nad4l*, *trnT*, *trnP* and *nad6*-*cob*-*trnS2*, and the present gene arrangement pattern may be derived from tandem duplications and random losses of these gene blocks. The gene order observed in *S. sinisalicis* mitogenome represents a new arrangement pattern that has never been discovered in any other aphid species, which may have important implications for understanding the evolution of aphid mitogenomes. It’s unclear whether this rearrangement is a mitochondrial signature for species of the subfamily Lachninae or an independent evolutionary event only for *S. sinisalicis*. A broader sampling of this subfamily will be needed to further investigate whether this new rearrangement pattern also occurs in other species.

Most aphids of other subfamilies possess a conserved gene order identical to that of ancestral insects. There are some exceptions with mostly involving the duplications, transpositions, reverse transpositions or reversals of some tRNA genes, such as several species from Fordini (Eriosomatinae) and Aphidinae (Figure 4). Notably, all species within the Fordini exhibit a transposition of *trnQ* and *trnM* except the *Baizongia pistaciae*, a basal species within Fordini. Whereas gene order of two Eriosomatini (Eriosomatinae) species is arranged in the same order as the ancestral insect. These indicate that the most recent common ancestor of Fordini aphids may have a typical arrangement of *trnQ*-*trnM*, which may be rearranged through an independent evolutionary event that occurred during subsequent species diversification of this tribe. In addition to the cases involving tRNAs, two rRNA gene reversals are found in *Aphis fabae mordvilkoi* from Aphidinae. Besides, a more prominent rearrangement including five tRNAs and one PCG is observed in *Pseudoregma bambucicola* (Hormaphidinae), with transpositions occurred within the gene cluster *trnG*-*nad3*-*trnA*-*trnR*-*trnN*-*trnS1* resulting in a novel arrangement pattern of *trnA*-*trnN*-*trnS1*-*trnR*-*trnG*-*nad3*, while the other two Hormaphidinae species have an ancestral gene order as most other aphids, suggesting that gene rearrangement in *P. bambucicola* seems to occur independently.

### 3.3. Protein Coding Genes and Codon Usage Patterns

The overall size (excluding stop codons) of 13 PCGs of *S. sinisalicis* was 10,923 bp, accounting for 63.8% of the total genome (Table 3). All PCGs showed a high A+T bias, ranging from 76.3% (*cox1*) to 91.2% (*atp8*). The AT-skew (−0.156) and GC-skew (−0.067) were both negative for the whole PCGs, reflecting a bias towards nucleotides T and C than their counterparts. The third codon position presented a much higher A+T content than that of the first and second codon positions, and tended to use T and C bases (Table 3). All protein-coding genes were initiated with the typical ATN codons, and terminated with TAA, except the *cox1* and *nad4* terminating with a truncated termination codon T, which is common in other metazoan mitogenomes [4,72]. These incomplete stop codons have been supposed to be completed through post-transcriptional polyadenylation [73].

**Figure 5:**
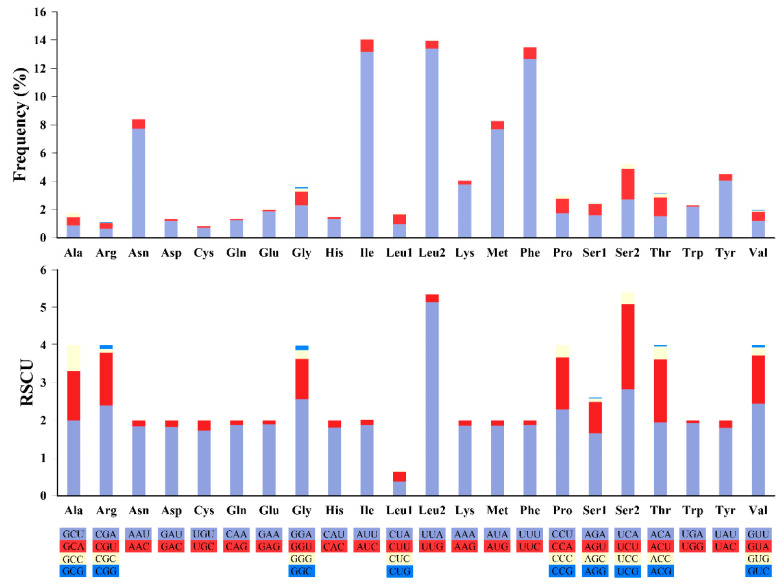
Codon usage in the mitochondrial genome of *Stomaphis sinisalicis*. The bar chart above indicates codon usage frequency of amino acids used for construction of 13 protein coding genes; and the bar chart below represents the relative synonymous codon usage (RSCU) of mitochondrial protein-coding genes. Codon families are labeled below the X-axis.

The codon usage analysis showed that the most frequently used codons were UUA-Leu2 (13.4%), AUU-Ile (13.2%) and UUU-Phe (12.7%), while the codons CUG-Leu1, UCG-Ser2, CCG-Pro and GCG-Ala were not used in the mitogenome (Figure 5, Table S2). The UUA (Leu2) also had the highest RSCU values, further indicating that UUA was the most preferred codon. The RSCU values of the PCGs revealed that there was a higher frequency in the usage of AT than that of GC in the third codon positions (Table S2).

### 3.4. Transfer and Ribosomal RNA Genes

**Figure 6:**
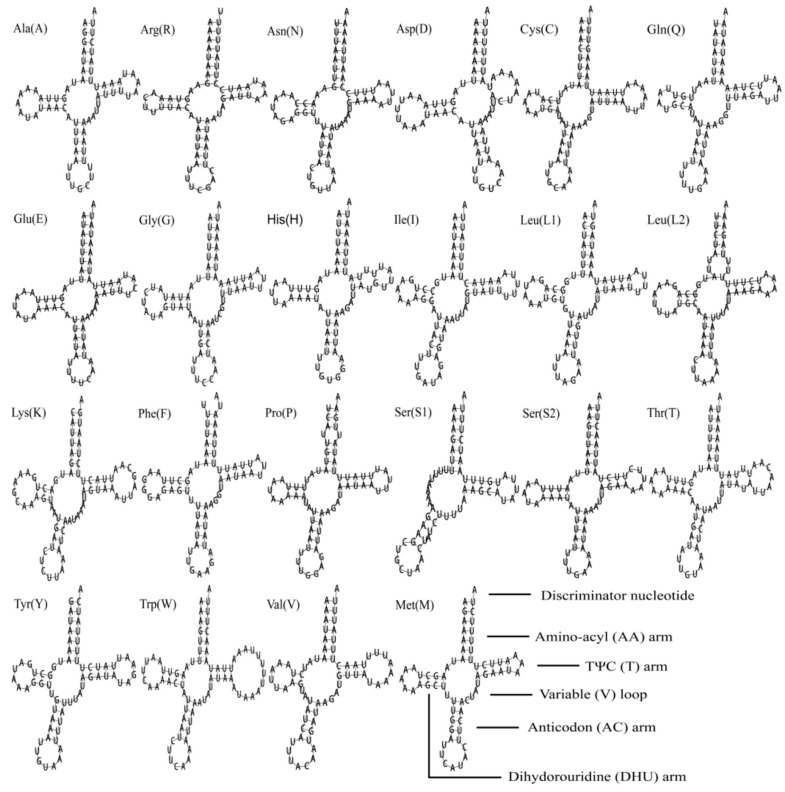
Secondary structures of the transfer RNA genes (tRNAs) in *Stomaphis sinisalicis* mitogenome.

The complete set of 22 typical tRNAs were all found in *S. sinisalicis* mitogenome, their secondary structures were shown in the Figure 6. The total length of tRNAs was 1478 bp, ranging from 60 bp to 75 bp in size. Both the AT-skew value (0.025) and GC-skew (0.170) were positively biased in the tRNAs, which showed a slight bias toward using A and an obvious bias toward G (Table 3). Most tRNAs exhibited the typical clover-leaf structures with two exceptions: *trnS1* missing the DHU arm and *trnD* without the TΨC stem. Lacking of DHU arm in trnS1 is a common feature for most metazoan mitogenomes [1]. This aberrant tRNAs are supposed to sustain their function via a posttranscriptional RNA editing mechanism [74,75].

The *rrnL* (1278 bp) and *rrnS* (777 bp) genes were located between *trnL1* and *trnV*, *trnV* and the control region, respectively. There was a little variation in the size of both rRNAs among different aphid species (Figure 2a). The overall rRNAs of *S. sinisalicis* mitogenome showed a negative AT-skew (−0.007) and a positive GC-skew (0.340), indicating a preference for using T base over A, and G over C (Table 3). The overall length and A+T content were rather conservative and identical to that of other aphid species.

### 3.5. Non-Protein Coding Regions

The AT-rich control region of *S. sinisalicis* mitogenome located between *rrnS* and *trnI*, spanning 919 bp in length. This is comparable to that of most other aphids, which have an average length of 867 bp (Table S1). Generally, the length of control region varies greatly with the size of embedded tandem repeats, which was not detected in the *S. sinisalicis* mitogenome. The control region is supposed to be involved in the initiation of replication and transcription of mitogenomes [76]. The control region in *S. sinisalicis* mitogenome had a higher A+T content (95.07%) compared to most aphid mitogenomes, which is varying from 81.75% (*Kaburagia rhusicola rhusicola*) to 95.07% (*P. bambucicola*). Both the AT-skew (−0.023) and GC-skew (−0.135) were negative, showing a bias towards using T and C (Table 3).

**Figure 7:**
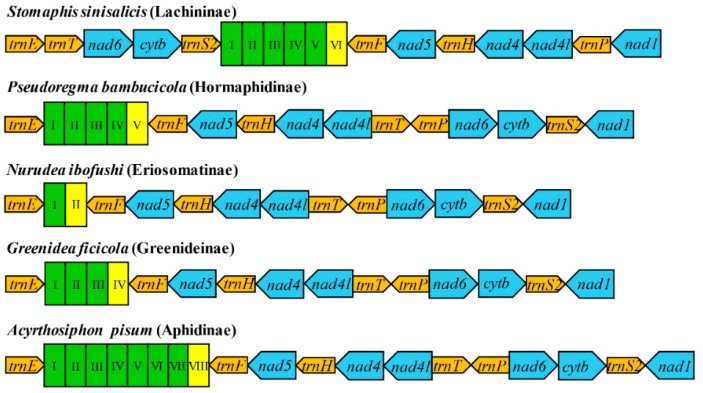
Repeat regions of the mitochondrial genome from *Stomaphis sinisalicis* and selected representative species from other aphid subfamilies. Green bars indicate tandem repeats, and the yellow bars refer to partial repeat unit; PCGs and tRNAs are indicated by the turquoise and orange squares, respectively.

In addition to the control region, a large non-coding repeat region (1489 bp) comprising five tandem repeat units ranging from 262 bp to 264 bp and a 171 bp partial repeat unit was also present in the *S. sinisalicis* mitogenome. Such special tandem repeats have been found in some aphid species from different subfamilies, and vary widely in size and number of repeat units, leading to varied lengths of repeat regions, ranging from 452 bp in *Nurudea yanoniella* to 2322 bp in *A. glycines*. However, in our analysis, obvious tandem repeats were not found in some aphid mitogenomes previously reported with repeat regions (*A. robiniae*, *A. fabae mordvilkoi*, *Myzus persicae*, *Rhopalosiphum nymphaeae* and *Sitobion avenae*; Table S1), indicating the validity of the annotation of repeat regions in these species need to be further verified. Figure 7 presents the repeat regions of *S. sinisalicis* and other representative species from four aphid subfamilies. Typically, the unique non-coding tandem repeat region found in known aphid mitogenomes from other subfamilies (Hormaphidinae, Eriosomatinae, Greenideinae and Aphidinae) is located between *trnE* and *trnF* [4,22,23,24], which has been thought an alternative origin of the mitogenome replication as in other insects [77]. However, the repeat region in *S. sinisalicis* mitogenome was found located between *trnS2* and *trnF*. According to the gene rearrangement analysis above, the repeat region along with the gene cluster *trnF*-*nad5*-*trnH*-*nad4*-*nad4l* may constitute a gene block, and go through the TRDL event together. The function of this unique tandem repeat region is currently unknown. However, the existence of repeat region in mitogenome of the basal species *S. sinisalicis* but not in *A. tsugae* may suggest that it is probably an ancient feature acquired by the common ancestor of Aphididae, which along with the scatter distribution within different subfamilies further favors the hypothesis about a single origin and subsequent diversification of the repeat region in aphid mitogenomes [5,6,25].

### 3.6. Phylogenetic Analysis

**Figure 8:**
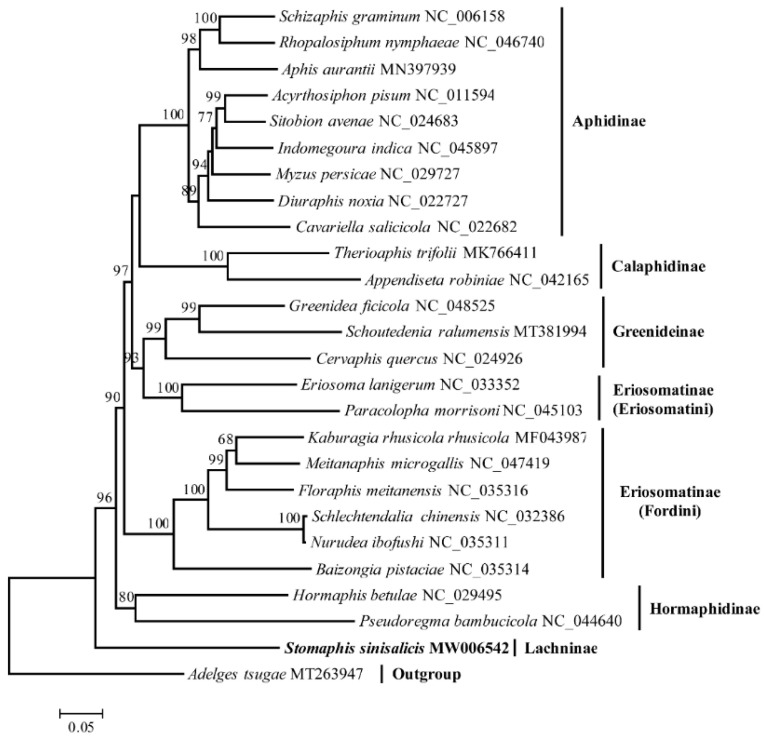
The maximum-likelihood phylogenetic tree based on 13 protein-coding genes of *Stomaphis sinisalicis* and 24 other aphids with the *Adelges tsugae* as outgroup. Bootstrap support values >50% are indicated above the branches.

The phylogenetic analysis using mitogenome data supported the monophyly of most subfamilies with high support values except the Eriosomatinae, which was found to be paraphyletic consistent with previous phylogenetic studies, with the Eriosoamtini clade clustering with the Greenideinae (Figure 8) [22,78]. The *S. sinisalicis* formed a separate clade locating at the basal position of Aphididae, which further confirms the previous proposed phylogenetic hypothesis [28]. This primitive taxonomic status makes this mitogenome an important data resource for future comparative studies to investigate the mitogenome evolution across different aphid lineages.

## 4. Conclusions

In this study, we reported the *S. sinisalicis* mitogenome, the first complete mitogenome from the subfamily Lachninae. The gene content, base composition and overall organization of mitogenome was analyzed in detail and compared to other aphid species. This mitogenome is similar to that of ancestral insect mitogenomes in terms of the gene content, base composition and overall organization; however, the gene order between the *trnE* and *nad1* is highly rearranged and differs greatly with other aphids. This gene rearrangement involving tRNAs, PCGs and the repeat region occurred in *S. sinisalicis* mitogenome has probably evolved from the ancestral gene order due to the tandem duplication and random loss events. Comparative analysis also indicates that the presence of repeat region may be an ancestral feature of aphid mitogenomes. Besides, phylogenetic analysis supports the basal position of Lachninae within Aphididae. Overall, our study provides valuable information for investigating the molecular evolution, mitogenome arrangement patterns and phylogenetic relationships across different aphid lineages.

## Figures and Tables

**Table 1:** Information for representative complete mitochondrial genomes of aphids used in this study.

Family	Subfamily	Species	Length (bp)	Accession Number	References
Adelgidae		*Adelges tsugae*	16,056	MT263947	[43]
Aphididae	Lachninae	*Stomaphis sinisalicis*	17,109	MW006542	This study
	Hormaphidinae	*Hamamelistes spinosus*	15,089	MT010853	[44]
		*Hormaphis betulae*	15,088	NC_029495	[45]
		*Pseudoregma bambucicola*	16,632	NC_044640	[24]
	Mindarinae	*Mindarus keteleerifoliae*	15,199	NC_033410	[20]
	Eriosomatinae	*Eriosoma lanigerum*	15,640	NC_033352	[21]
		*Paracolopha morrisoni*	16,330	NC_045103	[46]
		*Baizongia pistaciae*	15,602	NC_035314	[47]
		*Floraphis choui*	15,308	NC_035310	[48]
		*Floraphis meitanensis*	15,301	NC_035316	[48]
		*Kaburagia rhusicola ensigallis*	16,164	MF043984	[47]
		*Kaburagia rhusicola ovatirhusicola*	16,184	MF043985	[47]
		*Kaburagia rhusicola ovogallis*	16,164	MF043986	[47]
		*Kaburagia rhusicola rhusicola*	16,159	MF043987	[47]
		*Meitanaphis elongallis*	16,191	NC_035315	[48]
		*Meitanaphis flavogallis*	16,150	NC_035312	[48]
		*Meitanaphis microgallis*	16,191	NC_047419	[49]
		*Melaphis rhois*	15,436	NC_036065	[50]
		*Nurudea ibofushi*	16,054	NC_035311	[47]
		*Nurudea shiraii*	15,389	NC_035301	[47]
		*Nurudea yanoniella*	15,858	NC_035313	[48]
		*Schlechtendalia chinensis*	16,047	NC_032386	[51]
		*Schlechtendalia peitan*	15,609	NC_035302	[47]
	Greenideinae	*Cervaphis quercus*	15,272	NC_024926	[52]
		*Greenidea ficicola*	17,361	NC_048525	[22]
		*Greenidea psidii*	16,202	NC_041198	[6]
		*Schoutedenia ralumensis*	16,051	MT381994	[53]
	Calaphidinae	*Appendiseta robiniae*	15,049	NC_042165	[23]
		*Therioaphis trifolii*	16,068	MK766411	[54]
	Aphidinae	*Acyrthosiphon pisum*	16,971	NC_011594	NA
		*Aphis aurantii*	15,469	MN397939	[55]
		*Aphis citricidus*	16,763	NC_043903	[5]
		*Aphis craccivora*	15,308	NC_031387	[56]
		*Aphis fabae mordvilkoi*	15,346	NC_039988	[23]
		*Aphis glycines*	17,954	NC_045236	[25]
		*Aphis gossypii*	15,869	NC_024581	[57]
		*Cavariella salicicola*	16,317	NC_022682	[4]
		*Diuraphis noxia*	15,784	NC_022727	[7]
		*Hyalopterus pruni*	15,410	MT898422	NA
		*Indomegoura indica*	15,220	NC_045897	[58]
		*Myzus persicae*	17,382	NC_029727	[23]
		*Rhopalosiphum nymphaeae*	15,594	NC_046740	[59]
		*Schizaphis graminum*	15,721	NC_006158	[14]
		*Sitobion avenae*	15,180	NC_024683	[60]

Note: Only aphid species with complete mitogenomes and annotation information are listed; “NA” indicates sequences that are direct submission to GenBank.

**Table 2:** Features of the mitochondrial genome of *Stomaphis sinisalicis*.

Name	Strand	Location	Length (bp)	Start Codons	Stop Codons	Intergenic Sequence (bp)
*cox1*	J	1–1531	1531	ATA	T	−1
*trnL2* (taa)	J	1531–1599	67			−1
*cox2*	J	1599–2273	675	ATC	TAA	9
*trnK* (ctt)	J	2283–2354	72			3
*trnD* (gtc)	J	2358–2421	64			0
*atp8*	J	2422–2580	159	ATT	TAA	−20
*atp6*	J	2561–3214	654	ATT		4
*cox3*	J	3219–4004	786	ATG	TAA	−1
*trnG* (tcc)	J	4004–4070	67			0
*nad3*	J	4071–4424	354	ATT	TAA	−1
*trnA* (tgc)	J	4424–4490	67			8
*trnR* (tcg)	J	4499–4564	64			−3
*trnN* (gtt)	J	4562–4629	66			−2
*trnS1* (gct)	J	4628–4687	60			5
*trnE* (ttc)	J	4693–4760	66			34
*trnT* (tgt)	J	4795–4863	69			0
*nad6*	J	4864–5361	498	ATT		−1
*cytb*	J	5361–6473	1113	ATG	TAA	17
*trnS2* (tga)	J	6491–6557	65			7
repeat region	J	6565–8053	1489			6
*trnF* (gaa)	N	8060–8125	66			3
*nad5*	N	8129–9796	1668	ATA	TAA	50
*trnH* (gtg)	N	9847–9910	64			0
*nad4*	N	9911–11,219	1309	ATA	T	8
*nad4l*	N	11,228–11,521	294	ATG	TAA	24
*trnP* (tgg)	N	11,546–11,614	67			28
*nad1*	N	11,643–12,578	936	ATT	TAA	0
*trnL1* (tag)	N	12,579–12,643	65			6
*rrnL*	N	12,650–13,927	1278			0
*trnV* (tac)	N	13,928–13,994	67			3
*rrnS*	N	13,998–14,774	777			0
control region	J	14,775–15,693	919			0
*trnI* (gat)	J	15,694–15,759	66			−3
*trnQ* (ttg)	N	15,757–15,822	66			35
*trnM* (cat)	J	15,858–15,922	65			0
*nad2*	J	15,923–16,903	981	ATA	TAA	−1
*trnW* (tca)	J	16,903–16,977	60			−9
*trnC* (gca)	N	16,969–17,039	69			2
*trnY* (gta)	N	17,042–17,108	67			1

Note: Intergenic sequences refer to intergenic regions between each gene and the gene after it.

**Table 3:** Nucleotide composition of the *Stomaphis sinisalicis* mitogenome.

Regions	Size (bp)	T(U)	C	A	G	A+T (%)	G+C (%)	AT-Skew	GC-Skew
Whole genome	17,109	40.5	9.9	44.3	5.3	84.8	15.2	0.044	−0.305
PCGs *	10,923	48.3	8.8	35.2	7.7	83.5	16.5	−0.156	−0.067
1st codon	3641	40.3	8.7	40.4	10.6	80.7	19.3	0.001	0.098
2nd codon	3641	53.5	13.1	23.1	10.3	76.6	23.4	−0.397	−0.120
3rd codon	3641	51.1	4.6	42.3	2.1	93.4	6.7	−0.094	−0.373
tRNAs	1478	41.5	5.7	44.9	7.9	86.4	13.6	0.038	0.164
rRNAs	2055	42.9	4.9	42.2	10.0	85.1	14.9	−0.007	0.340
Repeat region	1489	41.5	9.7	44.4	4.3	85.9	14.0	0.034	−0.385
Control region	919	46.8	4.6	44.7	3.5	91.5	8.1	−0.023	−0.135

* Stop codons are excluded from the statistics of protein-coding genes.

## Data Availability

The complete mitochondrial genome of *S. sinisalicis* is available in the GenBank under accession number MW006542. The raw sequencing data are deposited in the NCBI Sequence Read Archive (SRA) database with accession number PRJNA668672.

## Supplementary Materials

The following are available online at https://www.mdpi.com/2075-4450/12/1/55/s1, Table S1: Information of aphid species with their representative complete mitochondrial genome sequences. Table S2: Codon usage of 13 PCGs in the mitogenome of *Stomaphis sinisalicis*.
