# Supplementary material for: The First Complete Mitochondrial Genome of Lachninae Species and Comparative Genomics Provide New Insights into the Evolution of Gene Rearrangement and the Repeat Region
Source: Insects. 2021 Jan 11;12(1):55. doi: 10.3390/insects12010055 (PMC7828084; doi:10.3390/insects12010055)
Supplement: Supplementary file 1 [file insects-12-00055-s001.zip › insects-1072056-supplementary_figures.docx]

Supplementary Figures


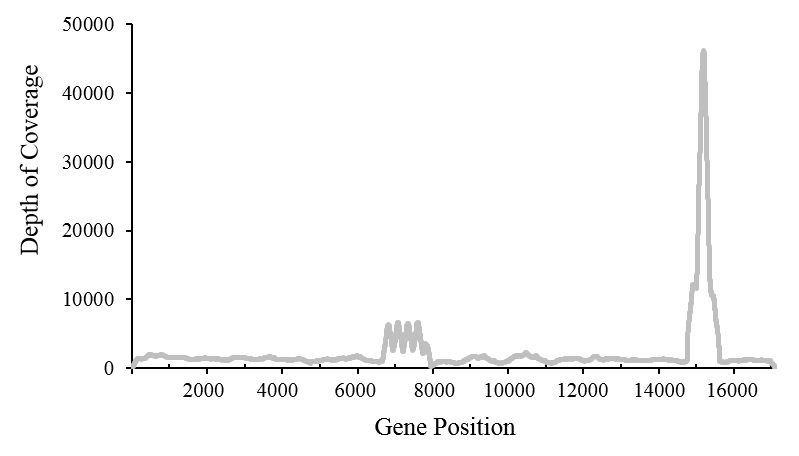


**Figure S1.** The per-position depth of coverage plot for the *Stomaphis sinisalicis* mitogenome. BWA software package and Samtools was used to map our raw data to the assembled genome sequence to compute the read depth for each position. Samtools coverage option in Samtools was then used to obtain a 100% sequence coverage for the genome sequence (with or without the repeat region) or the repeat region itself.


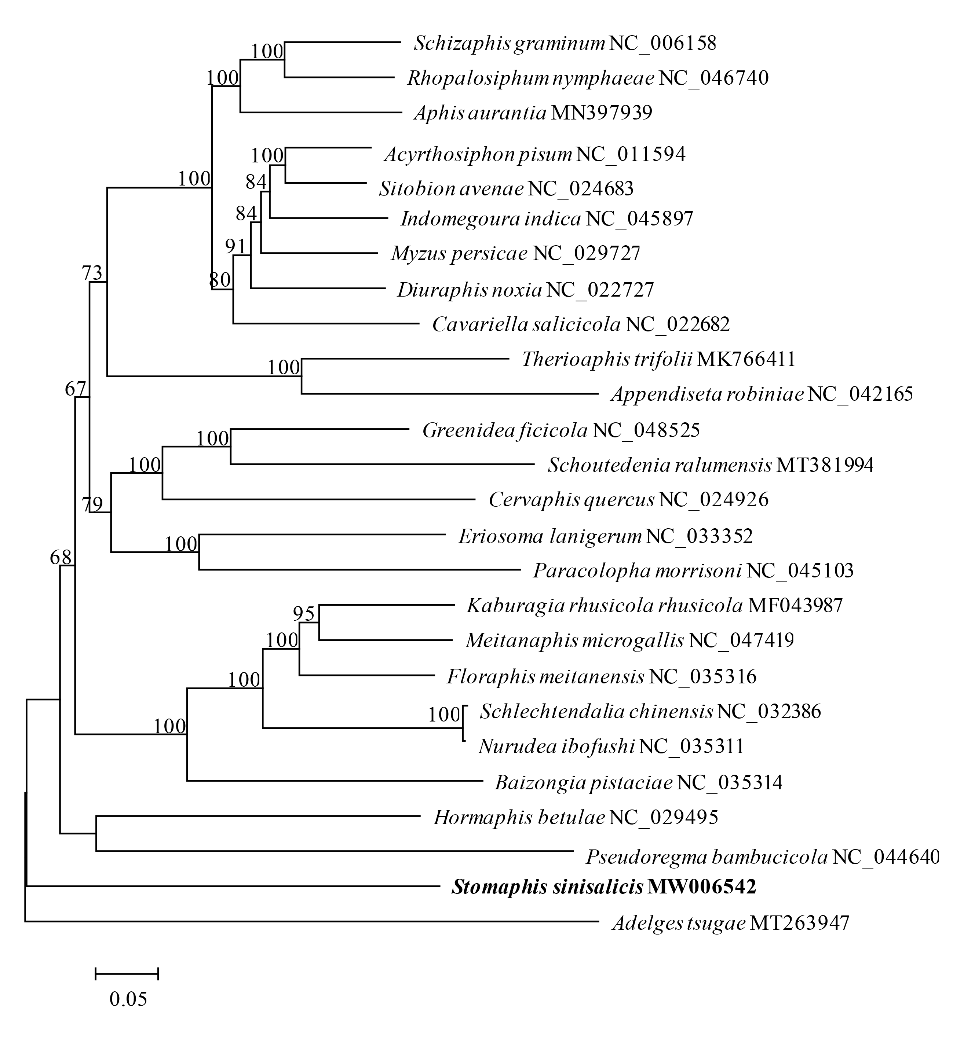


**Figure S2.** Phylogenetic tree based on the data matrix partitioned by each of the 13 protein-coding genes. Bootstrap support values > 50% are indicated above the branches.
